# Balloon Pericardiotomy: A Comprehensive Review and Case Series

**DOI:** 10.1016/j.jscai.2025.103800

**Published:** 2025-07-23

**Authors:** Jason B. Katz, Aravind Kalluri, Marysa Leya, Paul C. Cremer, Douglas R. Johnston, Mohamed Al-Kazaz, Daniel R. Schimmel

**Affiliations:** a Department of Medicine, Northwestern University Feinberg School of Medicine, Chicago, Illinois; b Division of Cardiology, Department of Medicine, Bluhm Cardiovascular Institute, Northwestern University Feinberg School of Medicine, Chicago, Illinois; c Division of Cardiology, Department of Radiology, Northwestern Memorial Hospital Bluhm Cardiovascular Institute, Northwestern University Feinberg School of Medicine, Chicago, Illinois; d Division of Cardiac Surgery, Northwestern University Feinberg School of Medicine, Chicago, Illinois

**Keywords:** balloon pericardiotomy, pericardial effusion, pericardiocentesis

## Abstract

Pericardial effusions can occur owing to a variety of reasons such as trauma, infection, autoimmune disease, and malignancy. Cardiac tamponade depends on the rate of fluid accumulation and not solely on the volume of the pericardial effusion. Rapid accumulation of pericardial fluid can lead to impaired cardiac filling and output with hemodynamic consequences, requiring urgent or emergent intervention. Despite initial intervention on patients with cardiac tamponade, recurrence of pericardial effusions has been estimated at approximately 20%, with a mean interval to recurrence of approximately 1 month. Both interventional and surgical techniques have been developed to relieve excess pericardial fluid including pericardiocentesis, surgical pericardiotomy, and percutaneous balloon pericardiotomy (PBP) with the latter 2 generally reserved for recurrent effusions. Rarely, surgical pericardiectomy is pursued. While safety and outcomes data are readily available for both pericardiocentesis and surgical pericardiotomies, PBPs are performed less frequently and at the few medical centers with the necessary expertise. In this case series, we present our center’s experience with PBP in the management of recurrent pericardial effusions in 4 different patients. We highlight their comorbidities and corresponding high surgical risk as well as review the technical considerations and outcomes of each patient. Aside from a small pneumothorax managed conservatively, there were no adverse side effects encountered. Balloon pericardiotomy is a safe and effective modality for pericardial effusion drainage in high-risk patients, which can improve patient comfort and hemodynamics.

## Introduction

Pericardial effusions can occur for a variety of reasons including trauma, infection, autoimmune disease, malignancy, and postinterventional cardiology procedures (ie, left atrial appendage closure, pacemaker and implantable cardioverter-defibrillator implantations, electrophysiological procedures, and structural interventions). Cardiac tamponade depends on the rate of fluid accumulation and not solely on the volume of the pericardial effusion. Rapid accumulation of pericardial fluid can lead to impaired cardiac filling and output with hemodynamic consequences, requiring urgent or emergent intervention. Despite initial intervention on patients with cardiac tamponade, recurrence of pericardial effusions has been estimated at approximately 20%, with a mean interval to recurrence of approximately 1 month.^1^

Both interventional and surgical techniques have been developed to relieve excess pericardial fluid including pericardiocentesis, surgical pericardiotomy, and percutaneous balloon pericardiotomy (PBP) with the latter 2 generally reserved for recurrent effusions.^2^ Rarely, surgical pericardiectomy is pursued. While safety and outcomes data are readily available for both pericardiocentesis and surgical pericardiotomies,^3^ PBPs are performed less frequently and at the few medical centers with the necessary expertise. Patients with iatrogenic hemorrhagic pericardial effusion postinterventional procedures are increasingly older with more serious comorbidities. Some develop recurrent pericardial effusions. Balloon pericardiotomy may also be considered as an alternative to surgical intervention for this group if the initial bleeding insult has been controlled and they are hemodynamically stable. In the acute setting, measurement of blood volume lost plus possible autotransfusion can be considered if a drain is in place. Balloon pericardiotomy should not be considered until the acute insult has been controlled. In this case series, we present our center’s experience with PBP in the management of recurrent pericardial effusions in 4 different patients (Table 1).

**Table 1:** Patient characteristics and outcomes undergoing percutaneous balloon pericardiotomy at our facility.

	Patient 1	Patient 2	Patient 3	Patient 4
Age, y	68	43	41	70
Sex	Female	Female	Female	Female
Comorbidities	Gastrointestinal stromal tumor (metastatic) Atrial fibrillation Left pleural effusion Hypertension Hypothyroid	Severe precapillary pulmonary hypertension secondary to Secundum ASD, bidirectional shunt Chronic hypoxemic respiratory failure Chronic right heart failure Asthma Gallstone pancreatitis Ischemic stroke (cardioembolic)	Severe idiopathic precapillary pulmonary hypertension Obstructive sleep apnea Obesity class III Hypertension Gout Asthma	Severe Group I pulmonary hypertension due to systemic sclerosis Interstitial lung disease Atrial fibrillation/atrial flutter
No. of previous pericardiocentesis	1	3	2	1
Indication	Recurrent large, symptomatic pericardial effusion causing right ventricular inversion s/p pericardiocentesis and drain before 2 mo Patient consent and medical frailty precluded surgical pericardial window	Recurrent large, symptomatic pericardial effusion causing right atrial systolic collapse s/p multiple previous pericardiocentesis	Recurrent large, symptomatic pericardial effusion with evidence of tamponade on cardiac MRI (right atrial systolic collapse and abnormal septal motion) s/p multiple previous pericardiocenteses	Recurrent large, pericardial effusion with tamponade s/p pericardial drain placement before 2 wk
Approach	Subxiphoid	Subxiphoid	Subxiphoid	Apical
Fluid analysis	Protein 2.3 g/dL Lactate dehydrogenase 540 U/L Negative cytology/bacterial/fungal/acid fast bacilli Cell count/differential: 461 nucleated cells/μL 11,100 RBC/μL 47% Macrophages 28% Lymphocytes 22% Neutrophils 3% Monocytes	Protein 5.1 g/dL Lactate dehydrogenase 218 U/L Negative cytology/bacterial/fungal/acid fast bacilli Cell count/differential: 800 nucleated cells/μL 895,000 RBC/μL 35% Lymphocytes 34% Neutrophils 28% Macrophages 3% Eosinophils	Protein 6.4 g/dL Lactate dehydrogenase 191 U/L Negative cytology/bacterial/fungal/acid fast bacilli Cell count/differential: 93 nucleated cells/μL 32 RBC/μL 85% Macrophages 7% Lymphocytes 6% Monocytes 2% Mesothelial cells	Protein/lactate dehydrogenase/cytology not done Negative bacterial/fungal/acid fast bacilli Cell count/differential: 138 nucleated cells/μL 24 RBC/μL 90% Macrophages 4% Neutrophils 3% lymphocytes 2% Mesothelial cells 1% Monocytes
Complications	None	None	None	Left apical pneumothorax (no chest tube required)
Long-term outcome	Passed on hospice care 1 mo later	Bilateral lung transplant with surgical ASD closure: Doing well on room air No pericardial effusion	Stable as outpatient on pulmonary vasodilators and diuretics Small pericardial effusion with no signs of tamponade	Doing well as outpatient on pulmonary vasodilators and diuretics No pericardial effusion 3 mo postdischarge

ASD, atrial septal defect; MRI, magnetic resonance imaging; RBC, red blood cell; s/p, status post.

## Background and outcomes

Percutaneous balloon pericardiotomy was first proposed as a therapeutic option by Palacios et al^4^ in 1991 for management of recurrent malignant pericardial effusion. In this initial article, all patients continued to have significant pericardial drainage 3 days after initial placement of pigtail drain, necessitating PBP. Since then, multiple case series have been published, detailing individual centers’ experiences with PBP.^5^, ^6^, ^7^, ^8^, ^9^, ^10^ Most, if not all, the patients included within these case series had malignant pericardial effusions, and PBP was generally pursued as it was considered a less-invasive alternative to surgical window. More recent case series have reported procedural success with balloon pericardiotomy for recurrent nonmalignant effusions, highlighting that this technique may also be applicable for patients for whom surgical intervention is not an option due to age and comorbidities.^11^

Previous series generally report high procedural success rates. The rare complications that have been reported have included fevers, pleural effusions requiring chest tubes or thoracenteses, pneumothorax, and pericardial bleeding.^7^ One case series of 50 patients reported technical success in 46, with short-term relief from recurrence of effusion but poor long-term prognosis with a mean survival time of 3.3 months.^7^ The largest of these registries (using only patients with malignant pericardial effusion) reported utilization of a second PBP in 15 patients after recurrence of initial PBP, with no reported recurrences after the second procedure.^12^ No significant difference in outcomes was noted in an observational study when PBP was delayed 2 days after emergency pericardial drainage compared with when it was performed at the time of initial pericardiocentesis.^10^ Finally, PBP as an initial intervention with no previous pericardiocentesis has also been established in a case series with 31 patients who underwent PBP as the index intervention.^5^

Although there are significant data comparing outcomes in pericardiocentesis versus surgical pericardial window,^3^ comparisons with PBP are lacking. Previous literature reviews suggested that surgical drainage of the pericardium may have improved symptom relief, decreased recurrence, and decreased morbidity when compared with nonsurgical approaches,^2^ although these data are largely limited by selection bias. In terms of surgical procedures, a pericardial window via video-assisted thoracoscopic surgery is increasingly being used since it has been described to be less traumatic than the anterior surgical approach and allows better visualization of pleural and pericardial structures, thus allowing a greater pericardial resection to allow greater drainage and facilitate drainage of loculated effusions.^13^ Nevertheless, 1 single-center study compared PBP with pericardiocentesis in malignant pericardial effusion found no statistical differences between reaccumulation rates (7.4% vs 14.3%, respectively) and complication rates (7.4% and 7.1%, respectively).^9^ Another systematic review reported recurrence rates of isolated pericardiocentesis of 38.3%, extended catheter drainage of 12.1%, and PBP of 10.3.^14^

Percutaneous balloon pericardiotomy is a therapeutic option for malignant recurrent pericardial effusions, particularly among patients in whom surgical pericardial window is not feasible due to prohibitively high surgical risk or potentially for patients who need pericardial effusion drainage for palliative measures. PBP has received a class IIB recommendation in the European guidelines for management of malignant pericardial effusion and cardiac tamponade.^15^

## Procedure

Percutaneous balloon pericardiotomy is performed by accessing the pericardial space, generally via subxiphoid approach in standard fashion for a pericardiocentesis (Central Illustration). Owing to distance from the skin to the pericardium, the parasternal approach is not amenable to balloon dilation of the pericardium. Similarly, the apical approach has the additional proximity of the pleura to the skin puncture site, potentially increasing risks for pneumothorax with PBP balloon dilation.

**Central Illustration:**
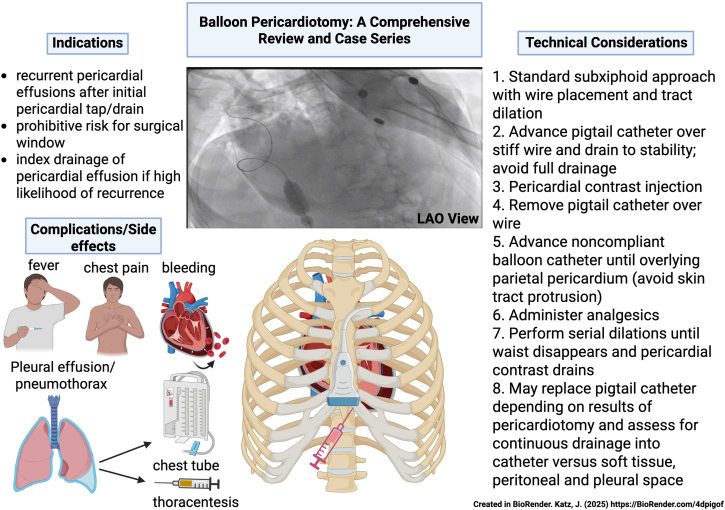
Balloon pericardiotomy is a percutaneous procedure that involves standard pericardial access followed by serial balloon dilations to create a pericardial defect allowing spontaneous drainage of fluid. It can be done for serial pericardial effusions in which surgical windows are not possible due to prohibitive operative risk or patient preference. Complications and side effects are listed above in addition to a left anterior oblique (LAO) angiographic view showing a wire in the pericardial space and real-time balloon dilation.

Once needle access to the pericardium via subxiphoid approach has been confirmed with echocardiographic or fluoroscopic guidance, a guide wire is introduced into the pericardium, the needle is removed, and the tract is dilated. Over a stiff wire, a pigtail drainage catheter can be placed, allowing drainage of fluid to correct any hemodynamic compromise from the effusion.

However, if the plan is to perform PBP, it is preferred that the effusion is not completely drained. With the pigtail catheter in place, a small amount of contrast can be injected into the pericardial fluid. This allows the operator to visualize the pericardial borders and center a dilation balloon. Over a stiff wire, the pigtail catheter is removed, and a long over-the-wire balloon catheter is advanced until the balloon overlies the parietal pericardium. It is important that the dilating balloon is not extending out of the skin tract. A steep left anterior oblique view may help identify balloon location. PBP may cause significant pain, but adequate intravenous analgesic immediately preceding inflation can often prevent significant discomfort. Subsequently, serial manual inflations are performed until the pericardial waist disappears. To obtain adequate dilation, the use of dilation balloons for aortic valvuloplasty has been described. In our experience, using a very noncompliant balloon, such as True Balloon (Medline) may be best for dilating and preventing recoil. A double-balloon technique has been described to maintain traction and improve durability.^16^ Traction and balloon manipulation may be necessary to prevent the balloon from shifting forward or backward.

The balloon size, length, and diameter can be chosen based on goals, patient comfort, and need for durability. If it does not seat well during the inflation, longer balloons (60 mm) will help the balloon expand over the target. However, balloon extending through the skin may cause discomfort. To help give strength to a compliant balloon or to assist in the balloon seating, a second (buddy) wire can be used, similar to the concept of a scoring balloon in the coronary or peripheral circulation. After needle access, if a sheath is placed, 2 wires can be inserted into the pericardial space with one to serve for balloon tracking and the other to serve as the second (buddy) wire for tracking and scoring the pericardium to assist in getting an adequate dilation in the pericardium.

Following these dilations, the balloon and guide wire are removed, allowing for continuous drainage from the pericardium into the mediastinal soft tissue, peritoneal space, and sometimes the pleural space. The peritoneum and pleura have a greater ability to accommodate the fluid production of the pericardial space. To ascertain the success of complete fluid removal, there are a number of tools that can be used. If a contrast injection was used to see the pericardial space, the operator will see the contrast drain out after inflation and deflation of the balloon. Reinsertion of the drain with final aspiration may be performed. But, the most thorough way to ensure the fluid is completely drained is completion of a bedside echocardiography before removal of the wire from the pericardial space.

## Case 1

A 68-year-old woman with a history of metastatic gastrointestinal stromal tumor (after multiple immunotherapies) and left pleural effusion presented with cardiac tamponade. Pericardiocentesis via parasternal approach was performed with removal 350 mL serosanguinous fluid and reduction in pericardial pressure from 14 to 1 mm Hg. Fluid analysis was negative for malignancy or infection. Her drain was removed after 1 day, and she was discharged on colchicine. The patient presented to the hospital 2 months thereafter with worsening fatigue, exertional dyspnea, and lower extremity edema. Transthoracic echocardiogram demonstrated reaccumulation of a large pericardial effusion measuring 1.8 cm at the right ventricular free wall echocardiographic evidence of impending tamponade. The patient declined surgical pericardial window but did not want to pursue hospice at that time, so PBP was performed.

After 200 mL of serous fluid removed, PBP was performed via subxiphoid approach with an 18-mm balloon. Serial inflations were performed with countertraction applied to further dilate the pericardiotomy. The patient tolerated the procedure well with no complications. The drain was removed 1 day later, with fluid analysis demonstrating no signs of malignancy or infection. Shortly thereafter, the patient was transitioned to home hospice care and expired 1 month after PBP.

## Case 2

A 43-year-old woman with a history of recurrent large hemorrhagic pericardial effusion, bidirectional atrial septal defect, and severe precapillary pulmonary hypertension on triple therapy presented with acute on chronic dyspnea and cough. Echocardiography revealed large circumferential pericardial effusion (5.5 cm posteriorly and 3.5 cm anteriorly in subcostal views) with evidence of tamponade physiology.

Following successful subxiphoid pericardiocentesis, removal of 225 mL of serosanguinous fluid was performed slowly to avoid hemodynamic decompensation in the setting of severe pulmonary hypertension and the patient underwent PBP the following week. During this procedure, a 0.035-inch J wire was inserted through the pre-existing pericardial drain and was exchanged for an Amplatz extra stiff wire (Boston Scientific) through a 7F dilator. A 20-mm Tyshak II balloon (BVM Medical) was then inserted into the pericardial space and was manually inflated multiple times until a waist disappeared, at which time the balloon was removed. The patient tolerated the procedure well with no complications, and the previous pericardial drain was removed. Fluid analysis from the previous pericardiocentesis with leave-in drain demonstrated a hemorrhagic, lymphocyte predominant fluid with no evidence of malignancy or infection. The patient experienced no additional recurrences of pericardial effusion within the following month, at which time she underwent uncomplicated bilateral lung transplantation and surgical pericardial patch atrial septal defect closure.

## Case 3

A 41-year-old woman with a history of group 1 pulmonary arterial hypertension, obesity, obstructive sleep apnea, asthma, gout, and hypertension presented for acute on chronic dyspnea and right ventricular failure in the setting of recurrent pericardial effusion. Patient had undergone successful pericardiocentesis approximately 3 months before. Repeat transthoracic echocardiogram showed a large (3.5 cm over left ventricle, 4 cm over right atrium) pericardial effusion with evidence of tamponade.

Owing to refractory symptoms despite aggressive diuresis, the patient underwent subxiphoid pericardiocentesis with removal of 200 mL of serous fluid. She underwent pulmonary artery catheter assisted diuresis for volume optimization before planned PBP. Using the previously placed pericardial drain as a guide, a straight tip Amplatz Super Stiff Guidewire (Boston Scientific) was passed into the pericardium. Multiple serial inflations with a 20-mm × 4-cm Tyshak balloon (BVM Medical), a 20- × 4.5-mm True Balloon (Medline) and a 22 × 4.5 cm True Balloon (Medline) were successful in dilating the pericardium. A pericardial drain was then placed back in the pericardial space and the remnant fluid drained manually. The drain was left in place and pulled 2 days later after transthoracic echocardiograms showed no evidence of cardiac tamponade, but moderate posterior and large lateral effusion remained. Pericardial fluid analysis showed a macrophage predominant serous fluid with no signs of malignancy or infection. However, on transthoracic echocardiogram, approximately 2 months postprocedurally, she was again noted to have enlargement to a large pericardial effusion adjacent to the left ventricle and right atrium but with no evidence of cardiac tamponade. This was serially monitored for 5 months postprocedurally. The effusion had reduced to a small size adjacent to the right atrium.

## Case 4

A 70-year-old woman with a history of group 1 pulmonary arterial hypertension secondary to scleroderma on quadruple therapy, interstitial lung disease, and atrial tachyarrhythmias presented with subacute dyspnea and acute on chronic hypoxemic respiratory failure. She was initially taken for pulmonary artery catheter-guided pericardiocentesis with leave-in drain due to large pericardial effusion with concern for tamponade. Her hospitalization was also notable for mixed right-sided cardiogenic and septic shock (*Streptococcus pneumoniae* pneumonia) requiring intubation and vasopressors.

**Figure 1:**
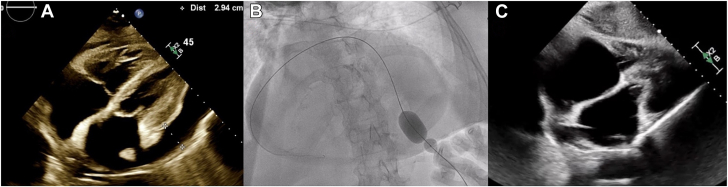
**Subxiphoid transthoracic echocardiogram before the procedure (A), percutaneous balloon pericardiotomy (B) and echocardiogram after (C) the procedure of patient 4.** Note the marked improvement in pericardial effusion posterior to the left ventricle.

After stabilization and transfer to the floor, she developed rapid reaccumulation of pericardial fluid (Figure 1A); the patient was taken for apical pericardiocentesis (safest approach given fluid pocket size) and balloon pericardiotomy. An Amplatz Super Stiff wire (Boston Scientific) was advanced through the micropuncture sheath and 8F dilator was used to dilate the tract. After inserting a new pericardial drain and confirming pericardial borders with contrast, a 10-mm Tyshak (BVM Medical), a 20-mm True Flow (Becton Dickinson), and Amplatz extra stiff balloon (Boston Scientific) were used for serial dilations (Figure 1B). The procedure was complicated by a small left apical pneumothorax, noted at the conclusion of the procedure. This was managed conservatively and self-resolved before discharge. A pericardial drain was left in place for 5 days due to retained contrast and concern for inadequate drainage. The patient was discharged with no major pericardial effusion on echocardiogram (Figure 1C) and echocardiogram 3 months postdischarge showed no pericardial effusion.

## Discussion and future directions

For a patient with malignant or recurring effusions of alternative etiologies, repeat pericardiocentesis versus balloon pericardiotomy are 2 options. There are no prospective data comparing the efficacy of initial balloon pericardiotomy to reduce the risk of requiring recurrent pericardiocentesis. Furthermore, there are no prospective data comparing the risks of an initial balloon pericardiotomy strategy over the risks and benefits of undergoing repeat pericardiocentesis. Further studies are required to address the efficacy and risks of using these 2 treatment strategies. However, a study of risks, benefits, and improvements of quality of life, comparing these 2 will be limited due to the infrequency of recurrent pericardial effusions and the specific patient population of patients with malignancy effusions having limited life expectancy.

For the patient in case 3, there was reaccumulation of a large effusion. This may be due to loss of outflow tract and lack of durability of a pericardiotomy procedure. On the contrary, it is possible, since the effusion was loculated and could not be completely drained at the initial presentation, that this was a risk factor for loss of the pericardial outflow tract.

## Conclusions

Pericardial effusions with hemodynamic compromise and the subsequent need for pericardiocentesis is a skillset used by interventional cardiologists for the rapid improvement in hemodynamics. Recurrent pericardial effusions occur with a frequency that requires additional treatment strategies to prevent repeated presentations of cardiac tamponade. Surgical pericardiotomy is the most performed strategy, but there are patients who benefit from balloon pericardiotomy, most frequently those at highest surgical risk and those with malignant pericardial effusions. With safe technique and attention to patient comfort, balloon pericardiotomy may reduce recurrence, repeated procedures, and hospitalizations.

## References

[1] Rafique A.M., Patel N., Biner S. (2011). Frequency of recurrence of pericardial tamponade in patients with extended versus nonextended pericardial catheter drainage. Am J Cardiol.

[2] Jama G.M., Scarci M., Bowden J., Marciniak S.J. (2014). Palliative treatment for symptomatic malignant pericardial effusion. Interact Cardiovasc Thorac Surg.

[3] Horr S.E., Mentias A., Houghtaling P.L. (2017). Comparison of outcomes of pericardiocentesis versus surgical pericardial window in patients requiring drainage of pericardial effusions. Am J Cardiol.

[4] Palacios I.F., Tuzcu E.M., Ziskind A.A., Younger J., Block P.C. (1991). Percutaneous balloon pericardial window for patients with malignant pericardial effusion and tamponade. Catheter Cardiovasc Diagn.

[5] Bhardwaj R., Gharib W., Gharib W., Warden B., Jain A. (2015). Evaluation of safety and feasibility of percutaneous balloon pericardiotomy in hemodynamically significant pericardial effusion (review of 10-years experience in single center). J Interv Cardiol.

[6] Galli M., Politi A., Pedretti F., Castiglioni B., Zerboni S. (1995). Percutaneous balloon pericardiotomy for malignant pericardial tamponade. Chest.

[7] Ziskind A.A., Pearce A.C., Lemmon C.C. (1993). Percutaneous balloon pericardiotomy for the treatment of cardiac tamponade and large pericardial effusions: description of technique and report of the first 50 cases. J Am Coll Cardiol.

[8] Jalisi F.M., Morise A.P., Haque R., Jain A.C. (2004). Primary percutaneous balloon pericardiotomy. W V Med J.

[9] Swanson N., Mirza I., Wijesinghe N., Devlin G. (2008). Primary percutaneous balloon pericardiotomy for malignant pericardial effusion. Catheter Cardiovasc Interv.

[10] Wang H.J., Hsu K.L., Chiang F.T., Tseng C.D., Tseng Y.Z., Liau C.S. (2002). Technical and prognostic outcomes of double-balloon pericardiotomy for large malignancy-related pericardial effusions. Chest.

[11] Sigusch H.H., Geisler W., Surber R., Schönweiß M., Gerth J. (2022). Percutaneous balloon pericardiotomy: efficacy in a series of malignant and nonmalignant cases. Scand Cardiovasc J.

[12] Rivero-Santana B., Jimenez-Valero S., Jurado-Roman A., Galeote G., Lopez-Fernandez T., Moreno R. (2024). The BALTO Registry: long-term results of percutaneous BALloon pericardioTomy in oncological patients. Catheter Cardiovasc Interv.

[13] Georghiou G.P., Stamler A., Sharoni E. (2005). Video-assisted thoracoscopic pericardial window for diagnosis and management of pericardial effusions. Ann Thorac Surg.

[14] Virk S.A., Chandrakumar D., Villanueva C., Wolfenden H., Liou K., Cao C. (2015). Systematic review of percutaneous interventions for malignant pericardial effusion. Heart.

[15] Adler Y., Charron P., Imazio M. (2015). 2015 ESC Guidelines for the diagnosis and management of pericardial diseases: The Task Force for the Diagnosis and Management of Pericardial Diseases of the European Society of Cardiology (ESC)Endorsed by: The European Association for Cardio-Thoracic Surgery (EACTS). Eur Heart J.

[16] Iaffaldano R.A., Jones P., Lewis B.E., Eleftheriades E.G., Johnson S.A., McKiernan T.L. (1995). Percutaneous balloon pericardiotomy: a double-balloon technique. Cathet Cardiovasc Diagn.

